# A Preterm Rat Model for Immunonutritional Studies

**DOI:** 10.3390/nu11050999

**Published:** 2019-05-01

**Authors:** Blanca Grases-Pintó, Paulina Torres-Castro, Mar Abril-Gil, Margarida Castell, María J. Rodríguez-Lagunas, Francisco J. Pérez-Cano, Àngels Franch

**Affiliations:** 1 Physiology Section, Department of Biochemistry and Physiology, Faculty of Pharmacy and Food Science, University of Barcelona, 08028 Barcelona, Spain; blancagrases@ub.edu (B.G.-P.); mtorreca29@alumnes.ub.edu (P.T.-C.); mariadelmar.abril@ub.edu (M.A.-G.); margaridacastell@ub.edu (M.C.); mjrodriguez@ub.edu (M.J.R.-L.); angelsfranch@ub.edu (À.F.); 2 Nutrition and Food Safety Research Institute (INSA·UB), 08921 Santa Coloma de Gramenet, Spain

**Keywords:** prematurity, suckling rat, intestinal permeability, phagocytosis, plasmatic immunoglobulin, adaptive immunity, innate immunity

## Abstract

Neonates are born with an immature immune system, which develops during the first stages of life. This early immaturity is more acute in preterm newborns. The aim of the present study was to set up a preterm rat model, in which representative biomarkers of innate and adaptive immunity maturation that could be promoted by certain dietary interventions are established. Throughout the study, the body weight was registered. To evaluate the functionality of the intestinal epithelial barrier, in vivo permeability to dextrans was measured and a histomorphometric study was performed. Furthermore, the blood cell count, phagocytic activity of blood leukocytes and plasmatic immunoglobulins (Ig) were determined. Preterm rats showed lower erythrocyte and platelet concentration but a higher count of leukocytes than the term rats. Although there were no changes in the granulocytes’ ability to phagocytize, preterm monocytes had lower phagocytic activity. Moreover, lower plasma IgG and IgM concentrations were detected in preterm rats compared to full-term rats, without affecting IgA. Finally, the intestinal study revealed lower permeability in preterm rats and reduced goblet cell size. Here, we characterized a premature rat model, with differential immune system biomarkers, as a useful tool for immunonutritional studies aimed at boosting the development of the immune system.

## 1. Introduction

The length of human pregnancy is about 37−44 weeks [1] and when the neonate is born before the 37th week of gestation the newborn is considered premature [2]. There is a wide variety of factors involved in preterm birth, such as the age of the mother, the use of assisted reproductive technologies or multiple gestations, among others [3]. Generally, a premature birth is associated with the newborn’s low weight, fragile skin [4], lower neurodevelopment [5], lungs not fully developed with an absence or insufficient production of surfactant [6], immature stomach with lower acidity [4], and higher intestinal permeability with a low microbiota diversity [7]. These deficiencies make the preterm neonate more susceptible to infections and a higher rate of mortality and morbidity in later life [8].

The higher predisposition to suffering infections is due to the immaturity of their immune system, in both innate and adaptive immunity, and in the inadequate communication between these two systems [9,10]. In reference to innate immunity, preterm neonates have a lower count of neutrophils and monocytes and a lower cytokine production compared to term infants [11,12]. The defense mechanism against viral infections through the cytotoxic activity of the natural killer (NK) cells is also reduced in preterm compared to full-term neonates [11]. Furthermore, prematurity also affects the adaptive immunity: these infants have leukopenia, and lymphopenia [12]. The reduced count of lymphocytes is accompanied by a decrease in the secretion of cytokines [13] and immunoglobulins (Ig) [2]. In addition, this immaturity not only affects the systemic immune system, but also intestinal immunity. Premature infants show increased intestinal permeability and decreased levels of secretory IgA and mucus, making newborns susceptible to intestinal diseases such as infections or necrotizing enterocolitis (NEC) [14,15]. 

Animal models are essential to further understand the mechanisms involved in these processes and to generate new advances in this area. In this context, studies using preterm animal models could be useful to increase our knowledge of the state of prematurity, and to explore new insights in the field of nutritional interventions in preterm neonates in order to counteract immunodeficiencies. To date, there are few preterm models in rodents, and most of these are only focused on the impact of prematurity on brain [16] or lung development [17] or NEC [18,19], but none study immune system maturation, which is especially affected in prematurity. To our knowledge, the only premature animal model studying immunity was performed in pigs [20]. However, when studying the immune system in early life experimental approaches, it is highly valuable to provide models in species in which there is a remarkable immunological immaturity at birth, as in rodents [21]. For example, the supplementation with a particular strain of bifidobacteria has shown to have a preventive effect on the experimental rat model of NEC [19]; however, its impact on immune system development in a preterm animal remains unknown.

Therefore, the aim of the present work was to establish a preterm rat model to determine biomarkers of innate and adaptive immunity affected by prematurity. Specifically, we focused on studying the blood cell count, phagocytic function of blood leukocytes, plasmatic Ig pattern, intestinal permeability and histomorphometry of the small intestine.

## 2. Materials and Methods

### 2.1. Animals

The study was performed with six pregnant Wistar rats from Janvier Labs (Le Genest Saint Isle, France), specifically, with two dams at 13 days of gestation (G13), two at G14 and two at G15. Animals were individually housed in cages and monitored daily. The animals were housed under controlled temperature and humidity conditions, in a 12:12 h light:dark cycle and were fed with chow and water *ad libitum* in the Faculty of Pharmacy and Food Science animal facilities (University of Barcelona). 

All the experimental procedures were performed according to the Guide for the Care and Use of Laboratory Animals. The study was reviewed and approved by the Ethical Committee for Animal Experimentation of the University of Barcelona (CEEA/UB ref. 148/18).

### 2.2. Experimental Design

We disposed of three types of pregnant dams (2 dams/each), depending on their gestational stage: G13, G14 and G15. The G14 pregnant dams were allowed to deliver naturally, at day 22 of gestation, and their litters used to the term group. The G13 pregnant dams were subjected to a caesarean section at day 21 of gestation—just one day before normal delivery—and their litters made up the preterm group. As this surgery was not compatible with the mother’s survival, the preterm pups were given to the surrogate dams (G15), who had delivered naturally two days before the caesarean surgery of the G13 dams. This fact is highly important to ensure that they have enough milk in their breasts to breastfeed preterm pups.

Litters were culled to 10 pups per lactating dam and they had free access to the nipples and rat diet during the 10 days of the study. Each group was made up of 40% male and 60% female pups. Suckling rats were weighed daily and the handling was done in the same time range to avoid the influence of biological rhythms. On the last day of the study, body length (nose−anus) was measured, and the body mass index (BMI), calculated as body weight/length^2^ (g/cm^2^), and the Lee index, calculated as ^3^√weight/length × 1000 (^3^√g/cm), were determined. 

### 2.3. Caesarean Intervention

To obtain premature pups, a caesarean section, which was performed at G21, was required. The procedure was based on the methodology described by Appleby and Towner [22] with some modifications. Dams, partially anesthetized, were immediately sacrificed by cervical dislocation. Immediately, the offspring were extracted one by one by hysterectomy. Thereafter, they were randomly distributed among the two surrogate mothers’ cages, which had previously been separated from their own offspring. It should be mentioned that, in this study, the viability of preterm rats was 100% and they were not rejected by the surrogate dam. 

### 2.4. Sample Collection and Processing

At day 10, each litter was divided into two: five pups were used to perform a histomorphometric study and permeability assay in the small intestine and the other five were used to analyze the blood cell count, the plasmatic Ig concentration and the phagocytic activity of leukocytes. Animals were intramuscularly anesthetized with ketamine (90 mg/kg) (Merial Laboratories S.A., Barcelona, Spain) and xylazine (10 mg/kg) (Bayer A.G., Leverkusen, Germany) in order to be exsanguinated. Then, the small intestine was collected, weighed, and measured. To perform the histomorphometric study, the intestine was washed with PBS (phosphate buffer saline, pH 7.2; 154 mM sodium chloride (NaCl), 3.99 mM sodium dihydrogen phosphate monohydrate (NaH_2_PO_4_·H_2_O) and 16 mM disodium hydrogen phosphate dehydrate (Na_2_HPO_4_·2H_2_O)) and 1 cm sections were cut from the distal jejunum, placed in cassettes and fixed in 4% paraformaldehyde for 24 h. Blood samples were collected in heparin/lithium tubes (Sarstedt, Nümbrecht, Germany). Part of the whole blood was obtained to study the phagocytic activity and the blood cell count. The remaining blood was centrifuged to obtain plasma for Ig quantification. To determine the composition of the cellular elements of the blood, an automated hematological analyzer (Spincell 3, Spinreact, Barcelona, Spain) was used.

### 2.5. Immunoglobulin Quantification

At the end of the study, plasmatic IgG, IgM and IgA concentrations were quantified using a rat IgG, IgM and IgA ELISA quantification set (Bethyl Laboratories, Montgomery, TX, USA), as performed in previous studies [23]. Absorbance was quantified using a microplate photometer (LabSystem Multiskan, LabX, Midland, CA, USA) and data were interpolated into standard curves using ASCENT version 2.6 software (Thermo Fisher Scientific, Barcelona, Spain). The lower limits of detection were as follows: 1 ng/mL for IgG; 1.95 ng/mL for IgM; and 1.95 ng/mL for IgA.

### 2.6. Phagocytic Function of Blood Leukocytes

Phagocytosis was measured with the commercial kit Phagotest^®^ (Glycotope, Biotechnology, Heidelberg, Germany) according to the manufacturer’s instructions. Briefly, heparinized blood was incubated with opsonized fluorescein isothiocyanate (FITC)-labelled *Escherichia coli* for 10 min at 37 °C. A control tube remained on ice. After 10 min, phagocytosis was stopped by placing the tubes into the ice. Then, non-phagocytized bacteria were eliminated with the addition of the quenching solution. After washing and centrifuging (250× *g*, 5 min, 4 °C), cells were resuspended and incubated in a solution for lysis of erythrocytes and fixation of the leukocytes (20 min, room temperature). Finally, after an additional centrifugation and wash, the staining of the leukocyte DNA was carried out with a propidium iodide solution. 

Analyses were performed using a Gallios^TM^ flow cytometer (Beckman Coulter, Miami, FL, USA) at the Scientific and Technological Centers of the University of Barcelona (CCiT-UB). All samples were assessed by FlowJo, version 10 software (Tree Star Inc., Ashland, OR, USA). Monocyte and granulocyte populations were gated according to their forward-scatter and side-scatter characteristics (FSC/SSC). Then, their green fluorescence histogram (FITC) was analyzed. The phagocytic activity was expressed as percentage of fluorescent cells (monocytes or granulocytes) in the particular population studied. The mean fluorescence intensity indicative of the extent of phagocyte efficiency was also quantified. 

### 2.7. Intestinal Permeability Assay

The evaluation of the intestinal epithelial barrier functionality was performed by an *in vivo* permeability assay to dextrans. Specifically, 4 kDa-dextran conjugated to FITC (Sigma-Aldrich, Madrid, Spain) in PBS at a dose of 500 mg/kg were orally administered to rats using low-capacity syringes (Hamilton Bonaduz, Bonaduz, Switzerland) adapted to oral 23-gauge gavage tubes (ASICO, Westmont, IL, USA), as previously described [23]. A reference sample pool obtained by animals given an equivalent volume of PBS was used to rule out autofluorescent background in each tissue sample itself.

The offspring were separated from their respective mothers 1 h before the administration, to allow gastric emptying and to avoid the interference of milk components with the dextran solution. Four hours after the administration, the animals were euthanized (ketamine (90 mg/kg)/xylazine (10 mg/kg)), plasma was obtained, diluted, and the fluorescence emission was analyzed by triplicate at an excitation wavelength of 490 nm, in the Modulus™ Microplate spectrophotometer (Turner Biosystems, Sunnyvale, CA, USA). 

### 2.8. Intestinal Histomorphometric Study

Fixed intestinal tissues were dehydrated by a growing gradient of ethanol, paraffin-embedded and cut into 5 μm sections using a microtome (RM2135, Leica, Wetzlar, Germany) at the CCiT-UB. Then, samples were mounted on Superfrost™ Ultra Plus slides (Thermo Fisher Scientific), as previously described [24]. The samples were deparaffinized and rehydrated by periodic acid-Schiff (PAS) staining. Briefly, intestinal tissues were stained in a 0.5% periodic acid (Sigma-Aldrich) solution for 5 min and washed in distilled water. Afterwards, sections were immersed in Schiff’s reagent (Sigma-Aldrich) for 7 min, washed in running tap water and counterstained with Mayer’s hematoxylin solution (Sigma-Aldrich) for 1 min and again washed in running tap water. Finally, the slides were mounted with coverslips using ProLong^TM^ Gold Antifade Mountant (Life Technologies, Carlsbad, CA, USA). The observation of the intestinal architecture was accomplished using a bright-field microscope at 100X and 400X (Olympus BX41, Olympus Corporation, Shinjuku, Tokyo, Japan). All the morphometric measurements were processed with the Image J. software (Image Processing and Analysis in Java, National Institute of Mental Health, Bethesda, MD, USA). The morphometric analysis was focused on intestinal villi, 6−10 villi per animal and *n* = 5 animals/group were evaluated. Specifically, the villi height, width, area and epithelium perimeter of the villi were measured. The villi height was measured from the base of the villi (junction of the villi to crypts) to the top of the villi. Moreover, the villi width was calculated at the middle of the villi. The epithelial perimeter of the villi was evaluated by measuring the outer layer of the villi. Goblet cells are expressed as PAS-positive. The number of goblet cells per villi were counted manually and their area was also measured. Statistical analysis was performed with the average values of each animal.

### 2.9. Statistical Analysis

Results are shown as means ± SEM. The software package SPSS 22.0 (PASW Statistics, SPSS, Chicago, IL, USA) was used for statistical analysis. The Shapiro–Wilk and Levene’s tests were applied to assess normal distribution and variance equality, respectively. When they coexisted, the parametric Student’s t-test to compare the means between both experimental groups was carried out. To evaluate the correlation between body weight and Ig concentrations, Pearson’s coefficient (ρ) was applied. Differences between groups were considered statistically significant for *p* values < 0.05. 

## 3. Results

### 3.1. Effect of Prematurity on Body Weight and Other Morphometric Variables

**Figure 1:**
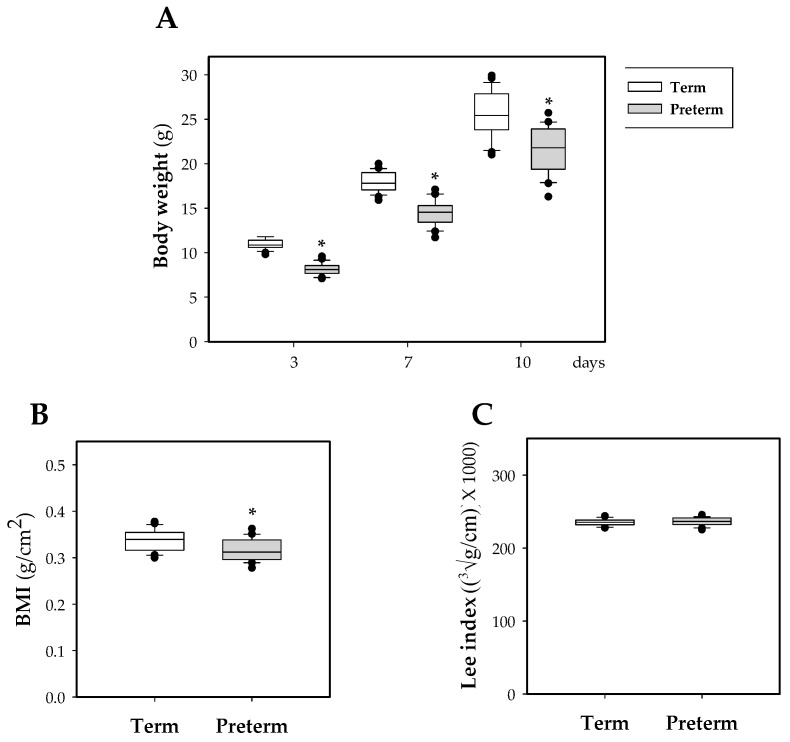
Box plot of the influence of prematurity on body weight and morphometric variables. Rat body weight along the study (**A**), BMI (**B**) and Lee index (**C**) at day 10 of life in both experimental groups. Line indicates the median, box shows the interquartile range (IQR) and the whiskers are 1.5 × IQR (*n* = 20 pups per group). Statistical differences: * *p* < 0.05 vs. term group.

Throughout the study, as expected, the weight of rats that were born in preterm conditions was lower than that of the term pups (*p* < 0.05, Figure 1A), indicating an important effect of the length of gestation period on body weight. This decrease has an impact on the BMI at day 10 (*p* < 0.01 vs. term group, Figure 1B). No differences were observed in the Lee index at the same day (Figure 1C).

Furthermore, to study in more depth the influence of prematurity on body weight variables, the weight of the spleen, thymus, liver, small and large intestines on day 10 was measured (Table 1). 

Preterm rats showed lower absolute weight in all these tissues than term ones (*p* < 0.05); however, the relative weights between the studied groups were similar (Table 1). Moreover, the length of both intestines was also measured on this day and the results showed that premature rats also had shorter intestines (*p* < 0.05 vs. term group, Table 1).

### 3.2. Effect of Prematurity on Blood Cell Count

At the end of the study, the preterm group showed a lower count of erythrocytes and platelets, but a higher count of leukocytes in comparison to the term group (*p* < 0.05, Table 2). Although there was no difference on the hematocrit (HCT) and the mean corpuscular hemoglobin (MCH), a tendency to decrease hemoglobin (Hb) concentration was observed in premature rats (*p* < 0.08 vs. term group). In addition, premature erythrocytes showed a slight macrocytosis through mean corpuscular volume (MCV), which was significantly higher than that in term rats (*p* < 0.05, Table 2). 

**Figure 2:**
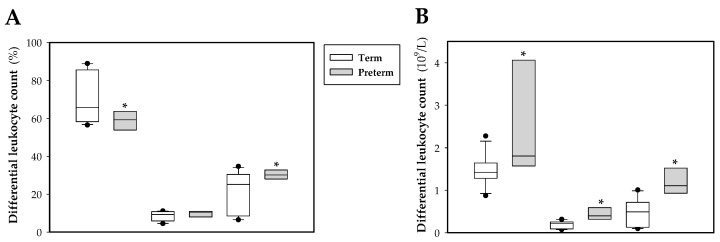
Box plot of differential leukocyte count in percentage (**A**) and in absolute values (**B**) at the end of the study (day 10) in both experimental groups. Line indicates the median, box shows the interquartile range (IQR) and the whiskers are 1.5 × IQR (*n* = 10 pups per group). Statistical differences: * *p* < 0.05 vs. term group.

Regarding the differential leukocyte count (Figure 2A,B), a tendency to decrease lymphocyte and increase granulocyte percentage in preterm animals was observed (*p* < 0.09 and *p* < 0.07 vs. term group, respectively), with no differences in monocyte percentage (Figure 2A). In absolute values, there was an increase in the count of lymphocytes, monocytes and granulocytes (*p* < 0.05, Figure 2B). 

### 3.3. Effect of Prematurity on Plasma Ig Concentrations

**Figure 3:**
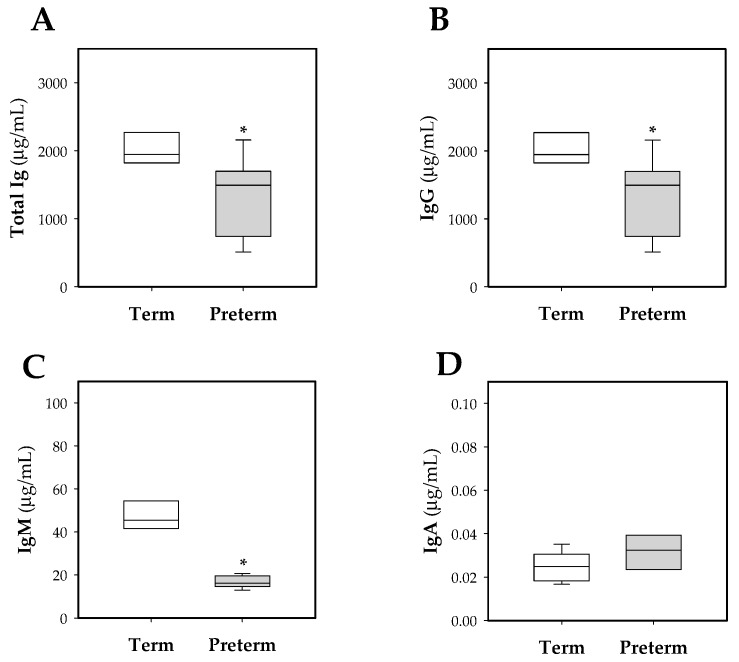
Box plot of plasmatic Ig concentrations at day 10 of life in both experimental groups. Total Ig (**A**), IgG (**B**), IgM (**C**), IgA (**D**). Line indicates the median, box shows the interquartile range (IQR) and the whiskers are 1.5 × IQR (*n* = 10 pups per group). Statistical differences: * *p* < 0.05 vs. term group.

To assess the impact of prematurity on the antibody levels in the systemic compartment, total Ig and IgG, IgM, and IgA isotypes were quantified in plasma samples at day 10 of life (Figure 3). Preterm rats had lower concentrations of total Ig compared to term ones (*p* < 0.01, Figure 3A). This change was mainly due to a reduction in IgG concentration (*p* < 0.01, Figure 3B), but IgM isotype was also decreased (*p* < 0.01, Figure 3C). No changes in IgA levels were observed (Figure 3D).

Considering all the pups in the study, there was a positive correlation between the concentration of total Ig, IgG and IgM, and body weight (ρ = 0.74, *p* < 0.001 at total Ig; ρ = 0.73, *p* < 0.001 at IgG; ρ = 0.73, *p* < 0.001 at IgM). Looking at this in the preterm group, there was a positive correlation between total Ig, IgG and IgA, and body weight (ρ = 0.71, *p* < 0.05 at total Ig; ρ = 0.70, *p* < 0.05 at IgG; ρ = 0.72, *p* < 0.05 at IgA), whereas in the term group this correlation was not observed.

### 3.4. Effect of Prematurity on Phagocytic Function of Blood Leukocytes

**Figure 4:**
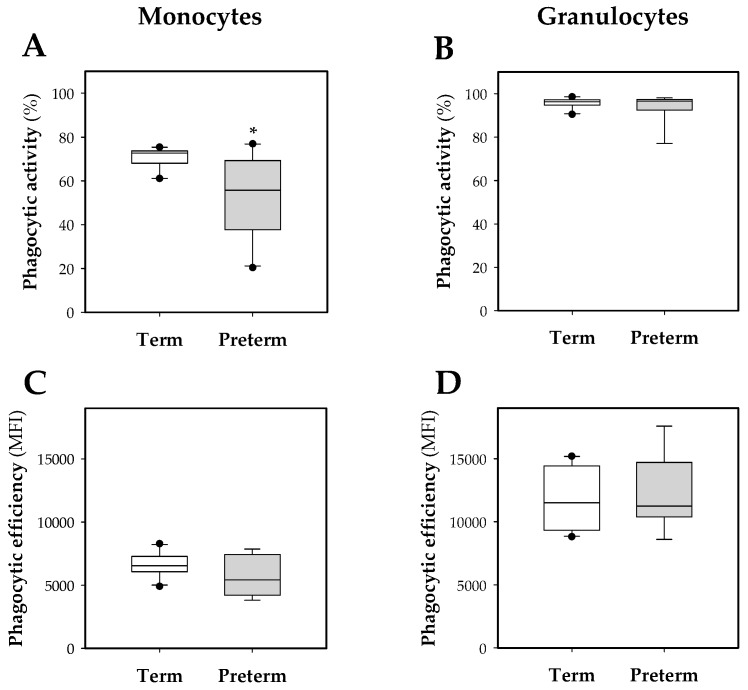
Box plot of phagocytic function of blood leukocytes. Phagocytic activity and efficiency in monocytes (**A**,**C**) and granulocytes (**B**,**D**). Line indicates the median, box shows the interquartile range (IQR) and the whiskers are 1.5 × IQR (*n* = 10 pups per group). Statistical differences: * *p* < 0.05 vs. term group.

Regarding monocyte phagocytic activity, this was around 70% in the term group and 60% in the preterm group, being lower in the latter (*p* < 0.05, Figure 4A). In contrast, the granulocyte population had a phagocytic activity higher than 90% without significant differences between either experimental groups (Figure 4B). 

The phagocytic efficiency of these leukocytes was also quantified (Figure 4C,D).The obtained results showed a higher phagocytic efficiency of the granulocyte population in comparison to the monocytes in both groups (*p <* 0.05); however, no statistical differences were found in the preterm group compared to the term group, either in granulocytes or in monocytes (Figure 4B,D).

### 3.5. Effect of Prematurity on Intestinal Barrier Function

**Figure 5:**
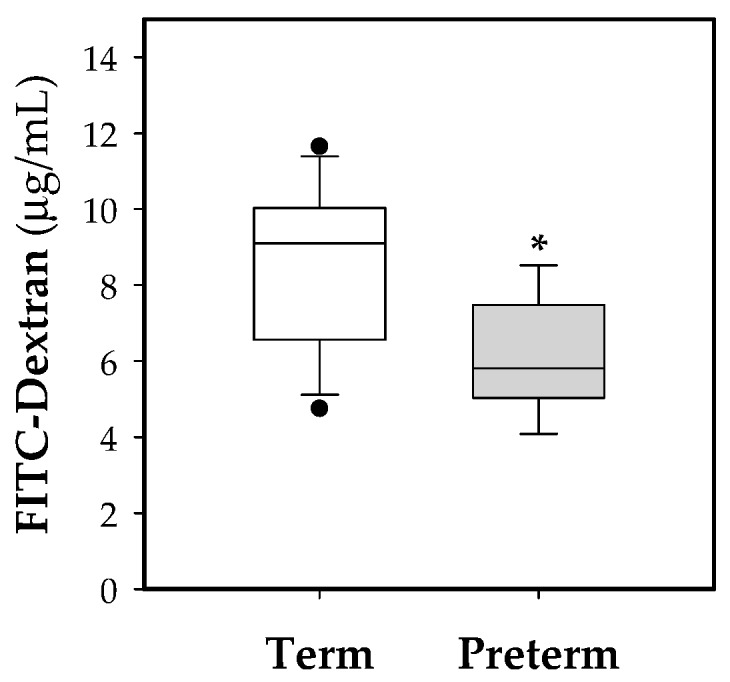
Box plot of intestinal permeability to 4 kDa-FITC-dextran in both experimental groups. Line indicates the median, box shows the interquartile range (IQR) and the whiskers are 1.5 × IQR (*n* = 10 pups per group). Statistical differences: * *p* < 0.05 vs. term group.

To study the intestinal epithelial barrier functionality, an *in vivo* permeability assay was performed to determine the paracellular transport of the 4 kDa-dextran labeled with FITC. Premature animals showed lower dextran-FITC signal in plasma than full-term ones, indicating lower permeability of the intestinal epithelium in preterm animals at 10 days old (*p* < 0.05, Figure 5).

### 3.6. Effect of Prematurity on Histomorphometric Characteristics of the Small Intestine

**Figure 6:**
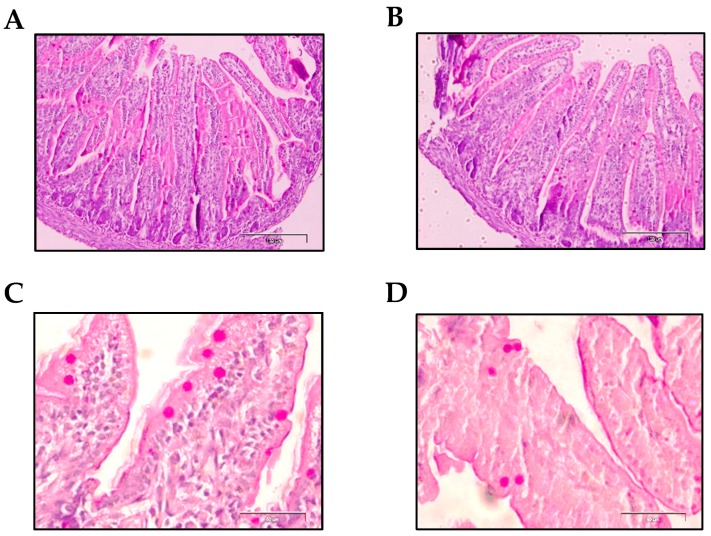
Representative images of histological sections of the jejunum with PAS staining. Term group (**A**,**C**) and preterm group (**B**,**D**). Goblet cells with densely stained granules can be observed along the length of the villi (**C**,**D**). Scale bar = 100 μm for 100X and 50 μm for 400X. Magnification = 100X (**A**,**B**) and 400X (**C**,**D**).

The morphology of the distal jejunum was studied, focusing on intestinal villi and goblet cell characteristics on day 10 of life. Table 3 shows the studied variables: villi width, villi height, villi area, villi perimeter, number of goblet cells per villi and goblet cell area. Histological differences due to prematurity were not found in any of the studied villi variables compared to the term group. With respect to goblet cells, although there was no difference in the number of these cells present in the villi, interestingly, preterm goblet cells had a smaller area than those found in term animals (*p* < 0.01, Table 3, Figure 6).

## 4. Discussion

The suckling rat born at term has been widely used as a model for immunonutrition studies in early life [21]. The effect of several dietary components on promoting the immature immune system development such as whey proteins [25], probiotics [26], adipokines [23,27], growth factors [28] or highly active immunomodulatory fatty acids such as conjugated linoleic acid (CLA) [29], have been studied. In addition, this rodent model has also been used in immunonutritional studies aiming to evaluate the protective action of prebiotics and probiotics during an acute intestinal infective process [24,30]. However, the impact of certain nutrients on the undeveloped response of the immune system in premature neonates has not been assessed, mainly due to the lack of optimal models to perform this kind of intervention.

The present study provides the set-up of a preterm rat model with the characterization of the immune state in the middle of the suckling period (day 10) with the purpose of establishing specific biomarkers of innate and adaptive immunity. For that, some variables have been assessed: blood cell count, plasmatic Ig, phagocytic function of blood leukocytes, intestinal permeability and intestinal morphometry. Nguyen et al. have established the development of the systemic immunity in a preterm pig model [20]. However, to the best of our knowledge, this is the first time that the effect of prematurity on the systemic and intestinal immune system in rat has been assessed. All these studies in preterm rats were compared with suckling rats born at term. 

It has been described that the length of the gestation period of rat is 19–22 days, whereas for pigs and humans it is 115 days and 280 days, respectively [31]. In this sense, it is estimated that in terms of translational relationship between age of rats and humans, 14 rat days are equivalent to 1 human year [32]. Thus, whereas the lymphoid architecture is formed prenatally in humans, it appears mostly postnatally in rats. Due to this, the development of the immune system in rats is delayed compared with humans, although both develop via similar schemes [21]. In addition, although the known differences between human and rodent immune responses, the uniform genetic background and the similarity to humans in terms of genetic and biochemical pathways led to an increasing interest in the suckling rat model as an approximation for immunonutritional studies. This is also the case in the context of early life and prematurity, where the short length periods of gestation and suckling in rats facilitate the nutritional intervention and in addition, as mentioned before, obtain a more immature pup than in larger species [21]. 

Although this work focuses on the study of the immune system, we also considered it necessary to evaluate some growth and body variables. Specifically, the body weight of the preterm group on day 3 of life was lower than that of those born at term, and this difference remained during the 10 days of study. Moreover, on day 10 of life, a lower BMI and organ weight were observed without affecting the relative weight or the Lee index. These results revealed the repercussions of prematurity during the first days of life. This feature found here in premature rodents is consistent with that previously found in humans. In fact, human preterm newborns also showed lower weight at birth [33]. In addition, at two years of age the incapacity to achieve similar weight values and BMI as those of term infants has been described [34]. Thus, the low weight of preterm rats observed in our study was not only similar to humans but also in line with other animal models, such as pigs [20]. 

Regarding the blood cell count, it was observed that prematurity causes an increase in the leukocyte count and a decrease in that of erythrocytes and platelets. Human studies focused on that point have evidenced that platelets showed an immature state not only in terms of a depletion of their count [35] but also in their functionality [27], thus, making preterm neonates more prone to hemorrhaging [36]. Focusing on erythrocytes, the mean corpuscular volume (MCV) was significantly higher in preterm rats and tended to decrease hemoglobin concentration in blood in comparison to the term group. These results taken together suggest a state of macrocytic anemia, and are in line with the study by Hoffbrand [37], which showed increased MCV due to a folic acid deficiency in preterm infants between 4 and 8 weeks old. This deficiency could be attributed to the fact that the preterm newborns require higher amounts of folic acid than those provided through diet intake and therefore leads to a depletion of their reserves. 

Besides changes in erythrocytes, the blood immune cell count was also modified due to prematurity. In this sense, it has been suggested that some features found in term rats due to its short gestation period, such as the intestinal barrier or the immune system immaturity at birth with respect to humans, can resemble those of infants born prematurely [21,31]. Thus, the present model aimed to show variables with even more immature immune levels. In fact, our results showed that the total blood leukocyte count in premature rats was much higher than those in the animals born at term. This result disagrees with previous studies in pigs and humans, which evidenced lower numbers of leukocytes in preterm compared to term newborns [12,20]. In our study, the preterm group’s differential leukocyte count showed an increase in the count of the three main populations (lymphocytes, monocytes and granulocytes). However, it has been described that preterm human babies had a lower number and percentage of granulocytes, monocytes and lymphocytes [2,11]. The results obtained in our model could be associated with a pro-inflammatory state interpreted as a defense mechanism against a highly hostile and aggressive environment for a premature newborn with a poorly developed immune system.

In this study, similar to what has been reported in humans, the preterm group had a lower Ig concentration compared to those born at term [2]. Moreover, the preterm group showed a positive correlation between the weight of the pups and total Ig, IgG, and IgA. In line with our results, it has been described that in human serum, the Ig concentration correlates directly with the gestational age and the weight of the baby at the time of birth [38]. In the case of IgG, low plasma levels found in the preterm group could be attributed to a deficient transplacental transfer of maternal IgG to the fetus, because it was interrupted after premature birth, knowing that the majority of maternal IgG is acquired during the last gestation period in humans and rodents [39,40]. However, in comparison with humans, who have a low postnatal transfer, rodents have an important postnatal transmission of IgG acquired from maternal breastmilk; thus, premature rats were able to improve this deficiency during the suckling period [40]. Regarding IgM, this Ig isotype does not cross the placenta in humans and rodents and, therefore, the IgM of the newborn comes basically from the endogenous synthesis [41,42]. The lower concentration of IgM found in premature rats could reflect the immaturity of B lymphocytes, both in their absolute cell count and their deficiency in Ig synthesis, compared to those born at term, as described previously in humans [2]. In relation to IgA, in humans it has been described that this isotype, as for IgM, does not substantially cross the placenta [43], but the intestinal balance of microbiota colonization of the newborn is essential for the stimulation of its production [44]. In addition, in human plasma, cells that produce IgA are absent at the intestinal level until the second month of life [45], and it has also been described that in rats the number of IgA- and IgM-secreting cells from intestinal lamina propria during the first 10 days is practically absent, and the number of IgA-secreting cells at the end of suckling period is far away from that of adults [21]. These facts explain the low plasmatic IgA concentration found in 10-day-old animals in both studied groups.

With regard to phagocytic activity, the percentage of granulocytes of 10-day-old animals was not modified in conditions of prematurity. On the other hand, compared to the term group, premature rats showed a lower percentage of monocytes exerting phagocytosis, which showed once again the immature status of the immune system in prematurity. These results, suggesting higher susceptibility to infections, are in line with other studies that report a lower phagocytic activity in monocytes in human infants or pigs that were born early in gestation [20,46]. Moreover, this could be related to the increase in the blood monocyte count, observed in this study, as a mechanism to compensate for this lack of phagocytic activity. In relation to the phagocytic efficiency of granulocytes, this was higher than that of monocytes, but did not differ between term and preterm groups, as previously described in humans [46,47]. Thus, this result is also in line with the translational potential of this preterm rat model to what happens in humans. In both rats and humans, this fact indicates that the phagocytic efficiency of leukocytes in relation to pathogenic bacteria is not affected by prematurity. 

In previous studies it has been shown that the intestinal barrier in rat is immature at the time of birth [48,49], and overall, in preterm humans as in other preterm animals, it has also been described that the intestinal epithelium has an increased permeability [14,43,50,51,52]. It is also known that this status of immaturity of the intestinal barrier is, in part, responsible for most cases of NEC in premature infants [53,54]. On this basis, in our study the effect of prematurity on the intestinal barrier was evaluated not only at the functional level, studying the permeability to 4-kDa-dextran, but also at the histomorphometric level, focusing on the study of the intestinal villi and goblet cells. Unexpectedly, in our work, a lower concentration of 4kDa-FITC-dextran in plasma was observed in conditions of prematurity, which indicates a lower permeability of the intestinal epithelium to this molecule in premature rats. However, in previous studies it has been observed that there is a rapid maturation of the intestinal barrier during the first days of life, positively influenced by feeding with breast milk, in both term [55] and preterm babies [56,57], thus reducing the incidence of NEC [58,59]. 

In addition, no differences between the groups were observed in the morphometric variables studied in the villi. However, goblet cells, responsible for the mucus layer found in the lumen of the intestine, were smaller due to prematurity. According to Clark et al. the smaller size and density of the goblet cells of the intestinal epithelium could reflect the condition of immaturity that would lead to lower mucus production at the intestinal barrier level [60]. This result was in line with other studies where the immaturity of these goblet cells is also described as being responsible for the deficient mucus layer [61]. Moreover, in those infants that suffer enterocolitis, immature goblet cells were also observed [53]. Again, this model resembles the conditions in preterm babies. 

This work has some experimental limitations, mainly due to the reduced number of dams and litters in each group. It was designed as a first approach considering the minimum use of animals following ethical standards; however, further nutritional studies should be conducted with a greater number of dams. Moreover, although we studied some variables related to the immune system, there are more immune variables to evaluate in a preterm model, such as the expression and localization of intestinal tight junctions, phenotype characterization of lymphocytes from different compartments (intestinal and systemic), functionality of lymphocytes (proliferation activity and the secretion of cytokines), activity of NK cells, intestinal microbiota, among others. Another limitation of the study was the mode of delivery: Term rats were born by vaginal delivery, whereas preterm rats were born by caesarean section, and this could be another variable influencing the difference between groups. This approach was required in order to obtain premature pups in a controlled manner without using inductor agents that could influence the final outcome. For future studies, one possibility in order to avoid the C-section and the influence of anesthesia, a caesarean could be performed in all groups, as performed by Stelloh et al. in mice [62]. In addition, it has to be taken into account that, in this model, preterm pups are being nourished with term milk. It is well known that there are remarkable differences between term and preterm milk such as higher levels of proteins, fat, and free amino acids, but there is a lower concentration of lactose in preterm milk [33]. In terms of immune factors, preterm milk is enriched in neutrophils, lactoferrin, cytokines (IL 6, IL-10, TNF-α), secretory IgA and growth factors [33,63]. This differential composition expressly stimulates the immature immune system of the preterm baby. Overall, this compensation through the dam’s milk has not been possible in this model, favoring a better separation of the results in the term and preterm variables studied.

There are several types of bioactive agents capable of stimulating the innate and acquired immunity of newborns and infants, such as some prebiotics, probiotics and symbiotics that lead to have a role in infection and allergy prevention [64,65,66]. However, few immunonutritional works have been performed so far in preterm neonates; the literature is scarce or even absent in terms of immunomaturation and they are just focused on NEC without considering immune variables. To date, some agents, for example, some *Bifidobacterium* strains and lactoferrin, have been demonstrated to significantly reduce the incidence of NEC and sepsis in preterm infants, but their impact on the premature immune system has not been evaluated [67,68]. Thus, the present model facilitates the study at preclinical level of the impact of bioactive agents able to promote the immune system development as a first step for future clinical interventions. The above interventions with immunonutrients target directly the pup after birth; however, the nutritional intervention to promote the maturation of the immune system of the preterm neonate can also be performed on gestating or lactating rats, as in previous studies in term rats [29].

In summary, our findings confirm the delayed development of innate and adaptive immunity in preterm rats compared with term ones. The premature rat model presented in this work is another step towards a better understanding of the immature immune system in premature conditions. In addition, we have described, for the first time, biomarkers of innate and adaptive immunity that could be useful in future studies to explore new ways to modulate this delayed immunity in preterm newborns, as well as being a useful tool in immunonutritional interventions. 

## Figures and Tables

**Table 1:** Absolute and relative weight of spleen, thymus, liver, small and large intestines, and length and relative length of small and large intestines from term and preterm groups at day 10.

	Term		Preterm	
	**Weight (g)**	**Relative Weight (%)**	**Weight** **(g)**	**Relative Weight (%)**
Spleen	0.14 ± 0.00	0.55 ± 0.01	0.12 ± 0.01 *	0.58 ± 0.03
Thymus	0.09 ± 0.00	0.34 ± 0.02	0.07 ± 0.00 *	0.33 ± 0.02
Liver	0.73 ± 0.03	2.82 ± 0.08	0.67 ± 0.03 *	3.08 ± 0.09
Small intestine	0.84 ± 0.03	3.29 ± 0.08	0.77 ± 0.03 *	3.51 ± 0.08
Large intestine	0.16 ± 0.01	0.63 ± 0.03	0.12 ± 0.01 *	0.57 ± 0.03
	**Length** **(cm)**	**Relative Length (%)**	**Length** **(cm)**	**Relative Length (%)**
Small intestine	41.04 ± 0.87	1.62 ± 0.03	37.20 ± 0.91 *	1.72 ± 0.05
Large intestine	6.88 ± 0.22	0.27 ± 0.01	6.19 ± 0.16 *	0.29 ± 0.01

The results are expressed as a mean ± S.E.M. (*n* = 20). Relative length percentage is expressed as cm of intestine per 100 g of animal body weight. Statistical differences: * *p* < 0.05 vs. term group.

**Table 2:** Blood cell count from both groups: term and preterm at day 10 of suckling period.

	Term	Preterm
Leukocytes (×10^9^/L)	2.18 ± 0.20	4.57 ± 0.79 *
Erythrocytes (×10^12^/L)	3.23 ± 0.08	2.83 ± 0.12 *
Hb (g/L)	77.58 ± 1.84	72.56 ± 1.89
HCT (%)	27.16 ± 0.79	25.01 ± 1.16
MCV (fL)	84.28 ± 1.30	88.42 ± 0.60 *
MCH (pg)	24.03 ± 0.42	25.82 ± 0.78
Platelets (×10^9^/L)	529.18 ± 33.41	408.38 ± 56.36 *

The results are expressed as a mean ± S.E.M. (*n* = 10). Statistical differences: * *p* < 0.05 vs. term group.

**Table 3:** Histomorphometric variables of small intestine: villi width, height, area, perimeter and the number of goblet/villi and their cellular area from term and preterm groups at day 10 of suckling period.

	Term	Preterm
Villi width (μm)	128.21 ± 15.91	173.54 ± 22.26
Villi height (μm)	602.03 ± 92.70	765.77 ± 136.41
Villi area (μm^2^)	75319.14 ± 22354.29	131378.51 ± 36991.03
Villi perimeter (μm)	1328.82 ± 191.19	1679.68 ± 278.57
Goblet cells/villi	6.79 ± 1.33	4.99 ± 0.44
Goblet cell area (μm^2^)	379.96 ± 26.72	239.08 ± 22.13 *

The results are expressed as a mean ± S.E.M. (*n* = 5). Statistical differences: * *p* < 0.05 vs. term group.
